# Supplementary material for: Characterization of the Growth and Morphology of a BSL-2 Coccidioides posadasii Strain That Persists in the Parasitic Life Cycle at Ambient CO2
Source: J Fungi (Basel). 2022 Apr 28;8(5):455. doi: 10.3390/jof8050455 (PMC9145405; doi:10.3390/jof8050455)
Supplement: Supplementary file 1 [file jof-08-00455-s001.zip › jof-1681058-supplementary.pdf]

Supplementary File S1– Sequence identity and peptide sequences of spherule-surface proteins.

C5P5N2_COCP7 (100%), 26,142.0 Da
Sod_Cu domain-containing protein OS=Coccidioides posadasii (strain C735) OX=222929 GN=CPC735_033580 PE=4 SV=1
10 exclusive unique peptides, 20 exclusive unique spectra, 36 total spectra, 49/248 amino acids (20% coverage)

```
MHAKSLLAFT SLLSAGFAAA QTGRLGDADV TVGNSPAVVY EAELLDKNNT
NFRGTVLVSG SSDGVGVIYN VNFTGFPPFG GPFLYHVHDQ PVPENGDCLG
TLAHLDPYER GEMPKCDPSR PQTCQVGDLS GKFGDIEGVN GSFSFQQQYH
DPYSSVTYGL GSFVGNRSIV VHFANTTRIN CGNFTLKEIR PGSRGLPCNG
RFCPNSPSGS MSVPVPTTTS PPTFEGDAAK VSVMSATGVL AALIGLLW
```

Peptide sequences: (R)LGDADVTVGNSPA(V); (R)LGDADVTVGNSPAVVY(E); (R)LGDADVTVGNSPAVVYEA(E); (R)GEMPKCDPSRPQTCQVGDLSGK(F); (K)CDPSRPQT(C); (K)CDPSRPQT(C); (K)CDPSRPQTCQ(V); (K)CDPSRPQTCQVGDLSGK(F); (C)DPSRPQTCQVGDLSGK(F); (R)INCGNFTLK(E);

Cu^{2+}/Zn^{2+}superoxide dismutase
NCBI Sequence ID ABB36775.1 (248 aa)

1 mhaksllaft sllsagfaaa qtgrlgdadv tvgnspavvy eaelldknnt nfrgtvlvsg
61 ssdgvgviyn vnftgfppfg gpflyhvhdq pvpengdclg tlahldpyer gempkcdpsr
121 pqtcqvgdls gkfgdiegvn gsfsfqqqyh dpyssvtygl gsfvgnrsiv vhfanttrin
181 cgnftlkeir pgsrglpcng rfcpnspsgs msvpvpttts pptfegdaak vsvmsatgvl
241 aaligllw

C5PIH6_COCP7 (100%), 26,378.8 Da
Uncharacterized protein OS=Coccidioides posadasii (strain C735) OX=222929 GN=CPC735_056990 PE=4 SV=1
5 exclusive unique peptides, 6 exclusive unique spectra, 6 total spectra, 42/231 amino acids (18% coverage)

```
MHLASILTCL LAATVSVSAS PGSERTQKRP SLAVPRCPRK ATASFDKSVP
EMKAFPNTQV DLCWEPTAFQ FTFKAFDETN FYFDPKHRTN DDIWKYEVME
AFIYHGTNDP QTYFEFEVSP NNVTYQTFVY NPSKVRKEGA PFDHFFVSDP
AADGFTSITT LDRKAQTWVS EVKIPLALFN VDRPRLSRWR MNFFRTVTSP
ATYPNQELGA WNSPDVASFH VTPFFGDVIL V
```

Peptide sequences: (R)KATASFDK(S); (K)AFDETNFYFDPK(H); (R)KAQTWVSEVK(I); (K)AQTWVSEVK(I); (K)IPLALFNVDRPR(L)

DOMON-like type 9 carbohydrate-binding module
NCBI Sequence ID AEB21190.1 (231 aa)

1 mhlasiltcl laatvsvsas pgsertqkrp slavprcprk atasfdksvp emkafpntqv
61 dlcweptafq ftfkafdetn fyfdpkhrtn ddiwkyevme afiyhgtndp qtyfefevsp
121 nnvtyqtfvy npskvrkega pfdhffvsdp aadgftsitt ldrkaqtwvs evkiplalfn
181 vdrprlsrwr mnffrtvtsp atypnqelga wnspdvasfh vtpffgdvil v

C5P9L1_COCP7 (100%), 43,558.2 Da
Aspartyl proteinase OS=Coccidioides posadasii (strain C735) OX=222929 GN=CPC735_005950 PE=3 SV=1
3 exclusive unique peptides, 3 exclusive unique spectra, 3 total spectra, 43/399 amino acids (11% coverage)

```
MRNSILLAAT VLLGCTSAKV HKLKLKKLPL TEQLEYGDIE THVRALGQKY
FGSLPSSQQQ TVLSDEYSTT GGHNVLVDNF LNAQYFSEIS IGNPPQNFKV
VLDTGSSNLW VPSSECGSIA CYLHNKYDSS ASSTYKKNGT EFAIRYGSGS
LSGFVSQDTL RIGDLTIEGQ DFAEATNEPG LAFAFGRFDG ILGLGYDTIS
VNKIVPPFYN MINEGLIDEP VFGFYLGDTN KEGDDSYATF GGVDSSLFSG
EMIKIPLRRK AYWEVDFDAI AFGNERAELE DTGIILDTGT SLIALPSTLA
ELLNREIGAK KSWNGQYTVD CNKRPSLPDL TFTLSGHNFT IGPYDYILEV
QGSCISSFMG MDFPEPVGPL AILGDAFLRR FYTMYDLGNN LVGLAKAGN
```

Peptide sequences: (K)YDSSASSTYKK(N); (R)YGSGSLSGFVSQDTLR(I); (R)FDGILGLGYDTISVNK(I)

Aspartyl proteinase
NCBI Sequence ID QVM08509.1 (399 aa)

1 mrnsillaat vllgctsakv hklklkklpl teqleygdie thvralgqky fgslpssqqq
61 tvlsdeystt gghnvlvdnf lnaqyfseis ignppqnfkv vldtgssnlw vpssecgsia
121 cylhnkydss asstykkngt efairygsgs lsgfvsqdtl rigdltiegq dfaeatnepg
181 lafafgrfdg ilglgydtis vnkivppfyn mineglidep vfgfylgdtn kegddsyatf
241 ggvdsslfsg emikiplrrk aywevdfdai afgneraele dtgiildtgt slialpstla
301 ellnreigak kswngqytvd cnkrpslpdl tftlsghnft igpydyilev qgscissfmg
361 mdfpepvgpl ailgdaflrr fytmydlgnn lvglakagn

C5PAF0_COCP7 (100%), 56,919.7 Da
alpha-1,2-Mannosidase OS=Coccidioides posadasii (strain C735) OX=222929 GN=CPC735_008870 PE=3 SV=1
2 exclusive unique peptides, 2 exclusive unique spectra, 2 total spectra, 28/519 amino acids (5% coverage)

M K G S P V L A V C   A A A L T L I P S V   V A L P M I D K D L   P S S I S Q S S D K   T S Q E R A E A V K
D A F R F A W E G Y   L E H A F P N D E L   H P V S N T P G N S   R N G W G A S A V D   A L S T A I I M D M
P D V V E K I L D H   I S N I D Y S Q T D   T M C S L F E T T I   R Y L G G M I S A Y   D L L K G P G S H L
V S D P A K V D V L   L A Q S L K L A D V   L K F A F D T K T G   I P A N E L N I T D   K S T D G S T T N G
L A T T G T L V L E   W T R L S D I T G D   P E Y G R L A Q K G   E S Y L L N P Q P S   S S E P F P G L V G
R T I D I E T G L F   R D D Y V S W G G G   S D S F Y E Y L I K   M Y V Y D K G R F G   K Y K D R W V T A A
E S T I E H L K S S   P S T R K D L T F V   A T Y S G G R L G L   N S G H L T C F D G   G N F L L G G Q I L
N R D D F T K F G L   E L V E G C Y A T Y   A A T A T K I G P E   G F G W D A T K V P   E A Q A E F Y K E A
G F Y I T T S Y Y N   L R P E V I E S I Y   Y A Y R M T K D P K   Y Q E W A W D A F V   A I N A T T R T S T
G F T A I G D V N T   P D G G R K Y D N Q   E S F L F A E V M K   Y S Y L I H S P E A   D W Q V A G P G G T
N A Y V F N T E A H   P V K V F S R G C

Peptide sequences: (R)TSTGFTAIGDVNTPDGGR(K); (Y)VFNTEAHPVK(V)

Mannosyl-oligosaccharide alpha-1,2-mannosidase
NCBI Sequence ID E9CXX8.1 (519 aa)

1 mkgspvlavc aaaltlipsv valpmidkdl pssisqssdk tsqeraeavk aafrfawegy
61 lehafpndel hpvsntpgns rngwgasavd alstaiimdm pdvvekildh isnidysqtd
121 tmcslfetti rylggmisay dllkgpgshl vsdpakvdvl laqslkladv lkfafdtktg
181 ipanelnitd kstdgsttng lattgtlvle wtrlsditgd peygrlaqkg esyllnpqps
241 ssepfpglvg rtidietglf rddyvswggg sdsfyeylik myvydkgrfg kykdrwvtaa
301 estiehlkss pstrkdltfv atysggrlgl nsghltcfdg gnfllggqil nrddftkfgl
361 elvegcyaty aatatkigpe gfgwdatkvp eaqaefykea gfyittsyyn lrpeviesiy
421 yayrmtkdpk yqewawdafv ainattrtst gftaigdvnt pdggrkydnq esflfaevmk
481 ysylihspea dwqvagpggt nayvfnteah pvkvfsrgc
